# Influences of Climate Change on Water Resources Availability in Jinjiang Basin, China

**DOI:** 10.1155/2014/908349

**Published:** 2014-02-17

**Authors:** Wenchao Sun, Jie Wang, Zhanjie Li, Xiaolei Yao, Jingshan Yu

**Affiliations:** ^1^College of Water Sciences, Beijing Normal University, Xinjiekouwai Street 19, Beijing 100875, China; ^2^Interdisciplinary Graduate School of Medicine and Engineering, University of Yamanashi, 4-3-11, Takeda, Kofu, Yamanashi 400-8511, Japan

## Abstract

The influences of climate change on water resources availability in Jinjiang Basin, China, were assessed using the Block-wise use of the TOPmodel with the Muskingum-Cunge routing method (BTOPMC) distributed hydrological model. The ensemble average of downscaled output from sixteen GCMs (General Circulation Models) for A1B emission scenario (medium CO_2_ emission) in the 2050s was adopted to build regional climate change scenario. The projected precipitation and temperature data were used to drive BTOPMC for predicting hydrological changes in the 2050s. Results show that evapotranspiration will increase in most time of a year. Runoff in summer to early autumn exhibits an increasing trend, while in the rest period of a year it shows a decreasing trend, especially in spring season. From the viewpoint of water resource availability, it is indicated that it has the possibility that water resources may not be sufficient to fulfill irrigation water demand in the spring season and one possible solution is to store more water in the reservoir in previous summer.

## 1. Introduction

Chinese economic boom in the past two decades brings a rapid increase in the demand of water resources. Annual water use rose from 522 billion m^3^ in 1993 to 611 billion m^3^ in 2011 [1]. Due to the impact of climate change and human activity, the uncertainty in water resource availability would likely be much higher in the 21st century [2]. Shortage of water resources is turning into a limiting factor to the sustainable economic development gradually in China [3]. It even happens in the humid area of China, due to intra-annual mismatch between water resources availability and consumption demand, especially in the spring season [4]. The Quanzhou City in Fujian Province, for which water supply highly relies on the surface water resources in Jinjiang Basin, is a typical area suffering from such mismatch. Two severe droughts have been reported in 2009 [5], which causes damages to the agriculture and industry. In such context, predicting hydrological changes under climate change is vital for making countermeasures against potential water resource shortage.

The objective of this study is to assess the impacts of climate change on the water resources availability in the Jinjiang Basin. The assessment about impacts of climate change on hydrological processes at basin scale usually involves building future regional climate scenario based on downscaled output of general circulation models (GCMs) and subsequently using the projected future meteorological data to drive hydrological models for making prediction. In this study, firstly, a grid-based distributed hydrological model Block-wise use of the TOPmodel with the Muskingum-Cunge routing method (BTOPMC) was applied to the Jinjiang Basin and calibrated based on streamflow data. Then the regional climate change scenario obtained from downscaled GCMs was used as the input of BTOPMC for predicting hydrological processes in the mid-21st century. At last, influences of climate changes on water resources availability in Jinjiang Basin were evaluated and some conclusions were drawn.

## 2. The Study Area

**Figure 1:**
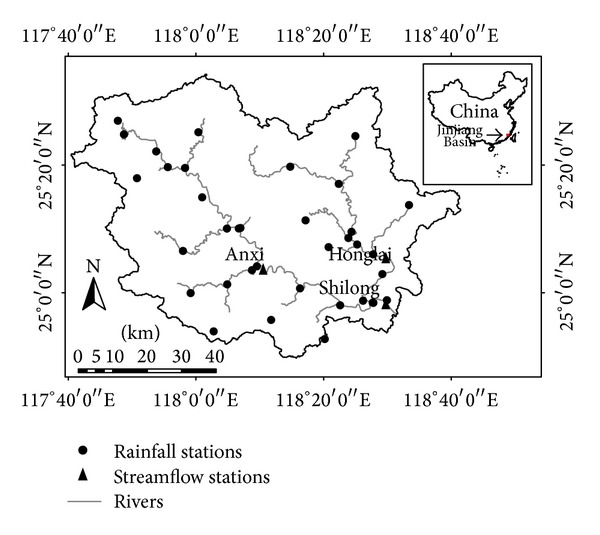
The river network, locations of streamflow stations and rainfall stations in Jinjiang Basin.

**Figure 2:**
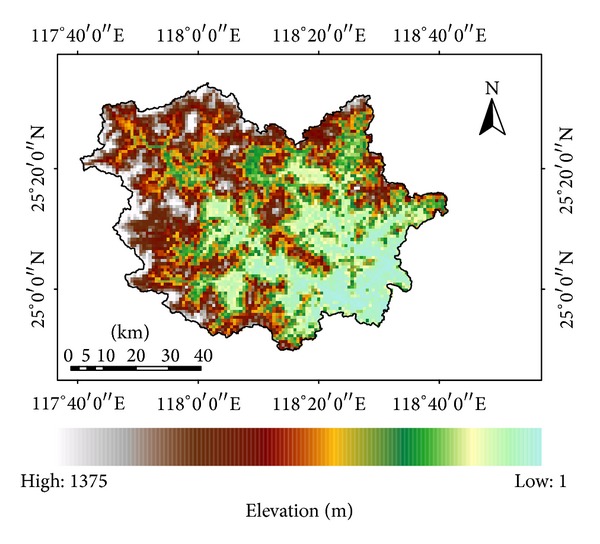
Topography of Jinjiang Basin.

Quanzhou is a coastal city in the Western Taiwan Straits Economic Zone of China. The whole area of the Jinjiang Basin is within the Quanzhou City. The river has two major tributaries (Figure 1), named as Xixi and Dongxi, which join together at Shuangxikou. The basin area is 5629 km^2^. It combines the mountainous area in the northwest and the low plain region in the southeast part of the basin. The elevation varies from 1 to 1375 m (Figure 2). The dominating land use types are forest and crop land. The basin has a subtropical monsoon climate characterized by dry winter season and rainy summer season. The annual precipitation ranges from 1000 mm to 1800 mm, for which 80% occurs in the period of March to September. The water intake infrastructure at Jinji sluice, which is located in several kilometers downstream the Shilong gauging station, makes major contributions to the water supply in Quanzhou City. The evaluation of water resources availability will be carried out based on the simulation of streamflow in the Shilong station.

## 3. The Hydrological Model

### 3.1. Model Description

The BTOPMC model is a physically based distributed hydrological model (see [6, 7] for details). The model has been successfully applied in many basins located in the Asian Monsoon area (e.g., [6, 8–10]). It divides the whole basin into many grid cells to account for spatial heterogeneity within the basin. At the same time, the grid cells are grouped into several blocks in order to keep the model structure in a relative simple manner. The runoff generation methodology is similar to TOPmodel [11]. The Muskingum-Cunge method [12] is employed for runoff routing. The BTOPMC considers saturation excess runoff, extending the TOPMODEL concept in a grid based framework for distributed hydrological simulation of large river basins.

**Figure 3:**
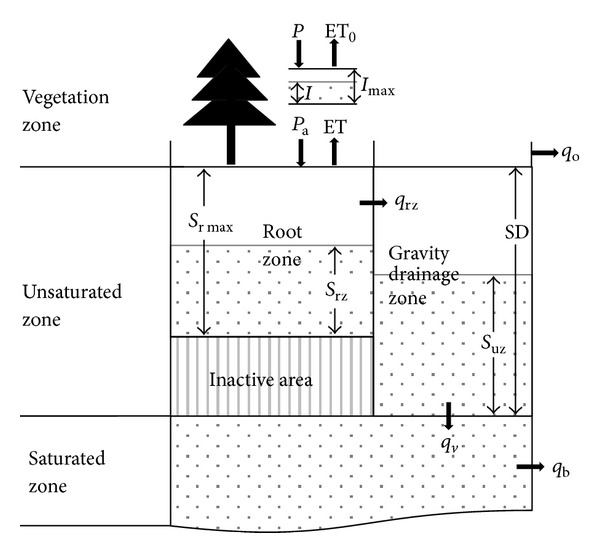
The schematic description of BTOPMC structure for runoff generation at each grid, where *P* is gross precipitation, *I*_max⁡_ is maximum interception capacity, *I* is interception state, ET_0_ is interception evaporation, ET is evapotranspiration from soil water, *S*_*r*max⁡_ is maximum root zone storage capacity, *S*_rz_ is soil water in root zone, *q*_rz_ is soil water moving from root zone to gravity drainage zone, *SD* and *S*_*uz*_ are soil water deficit and state in gravity drainage zone, *q*_*v*_ is groundwater recharge, *q*_*o*_ is overland flow, and *q*_*b*_ is base flow.

As shown in Figure 3, the vertical column of each grid includes vegetation zone, unsaturated zone, and saturated zone. The rainfall is first intercepted by the canopy in the vegetation zone for evaporation. Then the net rainfall is received by the unsaturated zone. In order to describe the complexity of hydrological processes in the unsaturated zone, it is further divided. The root zone and inactive zone represent the soil water storage between field capacity and wilting point. And only the portion in root zone can be used by vegetation. Net rainfall is assumed to infiltrate into the root zone until the field capacity is reached. The gravity drainage zone reflects the soil moisture between saturation and field capacity, which receives excess water from root zone. The overland runoff occurs when the soil water in the gravity drainage zone reaches saturation. The saturation zone receives recharge from gravity drainage zone and releases baseflow in a nonlinear manner. The potential evapotranspiration, either from the interception (PET0) or from the soil water in the root zone (PET), is computed based on the Shuttleworth-Wallace (S-W) model developed by Zhou et al. [15], for which only publicly available data are introduced, easing the application in data scarce basin. The evaporation from interception is calculated as the less of PET0 and canopy intercepted water amount. And the evapotranspiration from soil is the less of PET and water in root zone. The input data are tabulated in Table 1.

### 3.2. Model Setup and Calibration

The upstream area of Shilong gauging station is selected for BTOPMC application. The study area is divided into three blocks based on the location of Honglai and Anxi Gauging station. In total seven parameters need to be calibrated as listed in Table 2. The parameters values of each block were obtained through calibration based on the observed streamflow at the corresponding gauging station in each block. The calibration and validation periods are 2003-2004 and 2005–2007, respectively. The Nash-Sutcliffe Efficiency (NSE) and Volume Ratio (VR) are selected as model performance indicators:

(1) $$\begin{array}{c} \text{NSE}=1-\frac{\sum \left(Q_{\text{obs},i}-Q_{\text{sim},i}\right)}{\sum \left(Q_{\text{obs},i}-Q_{\text{obs},a}\right)} \end{array}$$

(2) $$\begin{array}{c} \text{VR}=\frac{\sum Q_{\text{sim},i}}{\sum Q_{\text{obs},i}}, \end{array}$$

where *Q*_obs,*i*_ and *Q*_sim,*i*_ are the observed and simulated streamflows at time step *i* and *Q*_sim,*a*_ is the average observed streamflow for the whole simulation period.

## 4. The Future Scenarios of Climate Change

The spatial scale of GCM output is too large to describe characteristics at basin scale; usually downscaling GCM is necessary [17]. Uncertainty of climate change projections from GCMs cannot be ignored [18, 19]. To make a more reliable future regional climate change scenario, usually an ensemble analysis combing multiple GCM analysis and quantifying the probability of future climate is conducted. Considering the above-mentioned issue, the Climate Wizard dataset (available at: http://www.climatewizard.org/index.html), projections of future temperature and precipitation derived from ensemble average of 16 GCMs, is adopted in this study. The monthly precipitation and mean temperature was downscaled to the resolution of 50 km using an empirical statistical method (see [20] for details). The data of the 2050s (2040–2069) for A1B scenario (medium CO_2_ emission) was used to build regional climate change scenario of the mid-21st century in Jinjiang Basin. The ensemble average of downscaled monthly mean temperature was directly used as input to compute potential evapotranspiration in the 2050s. The percentage changes of monthly mean precipitation between 2050s and historical climate (past 50 years) were used to scale daily precipitation data for 2005–2009. Then the projected precipitation data was inputted to BTOPMC. The BTOMPC simulation corresponding to this regional climate change scenario represents characteristics of hydrological processes in the 2050s. The simulation for the period from 2005 to 2009 is considered as the baseline. The differences between the two simulations are considered as the impact of climate change on hydrological system.

**Figure 4:**
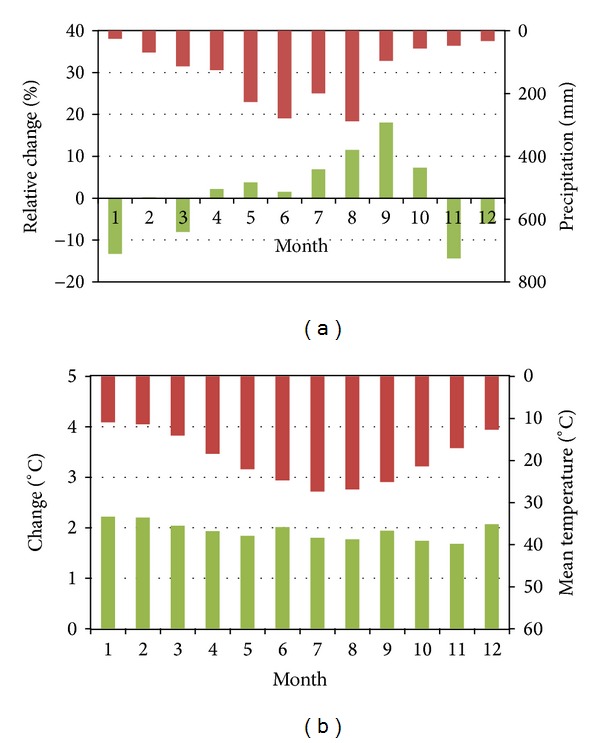
Basin averaged changes (green columns) of monthly mean precipitation and temperature between baseline (red columns) and future climate scenario in the 2050s.

Figure 4 demonstrates basin averaged relative changes of monthly mean precipitation and temperature between baseline and future climate scenario in the 2050s, respectively. The precipitation will increase from April to October. For the period from November to next March, the precipitation will decrease or remain almost the same with the baseline. For mean temperature in the 2050s scenario, it will increase with an average degree of 1.94°C in all of the twelve months.

## 5. Results and Discussion

### 5.1. The Performance of BTOPMC

**Figure 5:**
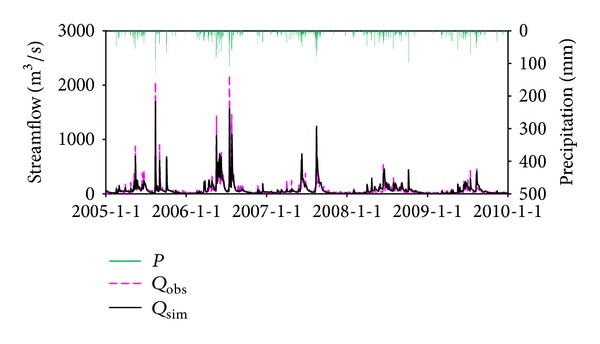
Streamflow simulations at Anxi station, where *P* is precipitation, *Q*_obs_ is observed streamflow, and *Q*_sim_ is simulated streamflow by BTOPMC.

**Figure 6:**
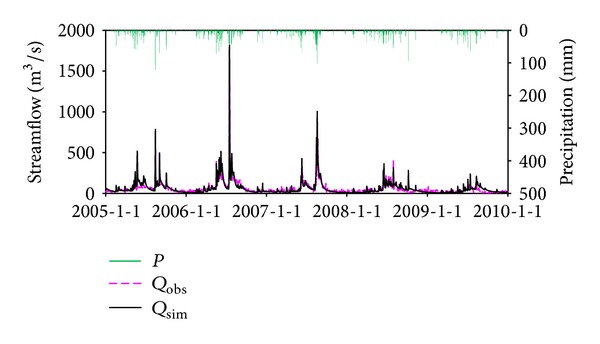
Streamflow simulations at Honglai station, where *P* is precipitation, *Q*_obs_ is observed streamflow, and *Q*_sim_ is simulated streamflow by BTOPMC.

**Figure 7:**
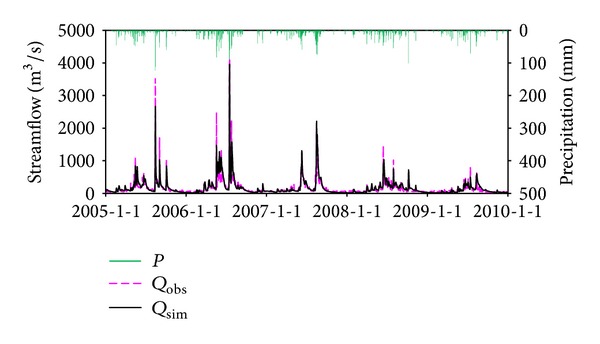
Streamflow simulations at Shilong station, where *P* is precipitation, *Q*_obs_ is observed streamflow, and *Q*_sim_ is simulated streamflow by BTOPMC.

**Figure 8:**
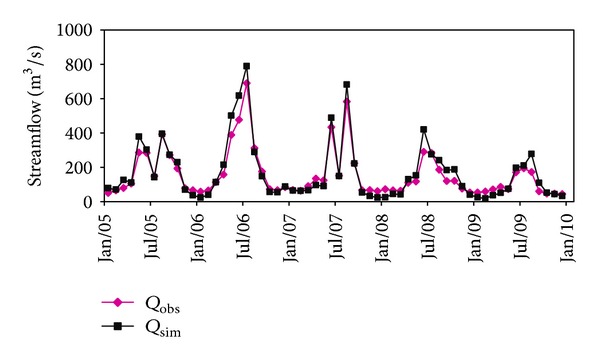
Comparison between monthly averaged simulated and observed streamflow at Shilong station.

The daily observed and simulated streamflow for the three gauging stations is shown in Figures 5, 6, and 7. It can be seen that the simulations can reproduce the timing and degree of streamflow variations fairly in each station. The values of NSE and VE for calibration and validation period are listed in Table 3, which indicates that simulation results are generally satisfying. The model performance in Honglai is lower than in the other two stations. One possible reason is that a reservoir named Shanmei is located in the upstream area of Honglai. However, due to the lack of the reservoir operation data, the impact of streamflow regulation was not considered in the simulation. As the most downstream station, the model performance at Shilong is slightly influenced by not considering the reservoir operation, which explains the simulation result at Shilong is worse than at Anxi (no reservoir in its upstream area) but better than at Honglai. As the impact of climate change will be explored at the monthly scale simulation at Shilong, the monthly simulated streamflow is also examined and demonstrated in Figure 8. The NSE is 93.1% for calibration period and 90.3% for validation period, respectively. The fairly well performance at both daily and monthly scales justifies the application of the calibrated model for the impact assessment being planned.

### 5.2. Influences of Climate Changes on Water Resources Availability

**Figure 9:**
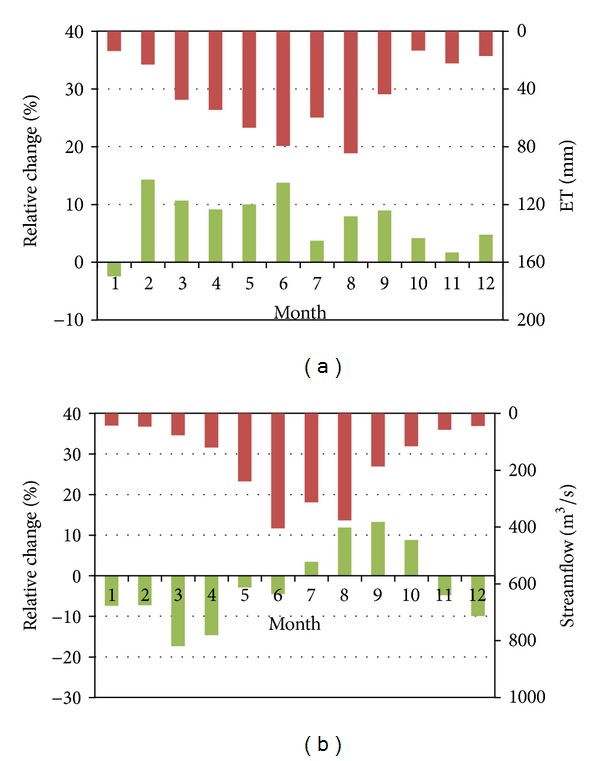
Basin averaged changes (green columns) of monthly mean ET and steamflow at Shilong station between baseline (red columns) and future climate scenario in the 2050s.

The daily meteorological data of 2050s A1B scenario, which was generated from downscaled ensemble prediction of 18 GCMs, were used as input of BTOPMC model for simulating hydrological processes in the future. The monthly relative changes of ET and runoff between the current situation (simulation from 2005 to 2009) and 2050s are depicted in Figure 9. The ET increases in the 2050s for all months, expect January. As mean temperatures in all months are higher in the 2050s, more energy is available for driving soil water and intercepted water to evaporate or transpire, which is the most probable reason of ET increasing in the eleven months. Meanwhile, the reduction of ET in January for 2050 scenario is considered as an outcome of sharp decrease of rainfall (13.3%), which lowers the availability of water for evapotranspiration.

The runoff in the 2050s exhibits an increasing trend in summer to early autumn (July, August, September, and October), with degrees varying from 3.4% (July) to 13.2% (September). This can be explained as a consequence of increasing rainfall in the same period. From November to June, the runoff in all of the eight months reduces compared with the current situation. For November, December, January, and March, it may result from the decreasing trend in rainfall. Due to the cumulative effects of rainfall reduction in previous months and higher ET, runoff in March decreases most, with an extent of 17.4%. These effects could also work as the explanation of why the runoff decreases in February, April, May, and June, even if rainfall increases or remains almost the same with current situation.

From the viewpoint of water resource availability, it is demonstrated that especially in the spring season, available water resource decreases in the 2050s A1B scenario, which may lead to the situation that there may be not sufficient water to fulfill irrigation water demand and consequently agricultural drought may happen. Since the prediction indicates that runoff will increase in summer seasons, one possible solution to the water deficit in the spring season is that, on the precondition avoiding flood disasters, the reservoir should store more water in previous summer for irrigation use in the spring.

## 6. Conclusion

For the purpose of evaluating the influences of climate change on water resources availability in Jinjiang Basin, the BTOPMC hydrological model was applied to simulate the hydrological processes in the basin. By comparing the simulation with observed streamflow data, it is indicated that the model can make fairly reasonable streamflow estimation, justifying using it for hydrological prediction. After constructing regional climate change scenario in the basin, the projected meteorological variables were inputted to BTOPMC model for predicting hydrological processes in the 2050s. The prediction shows that the basin may face water deficit in the spring season and one possible solution is to store more water in the reservoir in previous summer. The results of this study may be valuable for making reasonable water resource management policy in the Jinjiang Basin. To make the policy in a more quantitative manner, an analysis about the amount of water needed for irrigation is needed to decide how much extra water should be stored in the summer season.

## Figures and Tables

**Table 1:** Data used in BTOPMC modeling in Jinjiang Basin.

Type	Description	Remark
Physical data	Digital elevation map (DEM)	From GLOBE data, 30 arc second resolution Available at: http://www.ngdc.noaa.gov/mgg/topo/globe.html
	Land cover map	From IGBP classified global data, 1 km resolution Available at: http://edc2.usgs.gov/glcc/globdoc2_0.php
	Soil map	From harmonized world soil database (FAO/IIASA/ISRIC/ISSCAS/JRC, 2012) [13] 30 arc second resolution
	Soil texture	From harmonized world soil database (FAO/IIASA/ISRIC/ISSCAS/JRC, 2012) [13]

Meteorological data	Daily precipitation	From Bureau of Water Resources of Quanzhou City
	Monthly precipitation	From CRU 2.0 data set, input for the S-W model Available at: http://www.ipcc-data.org/obs/get_30yr_means.html
	Monthly cloud cover	From CRU 2.0 data set, input for the S-W model, Available at: http://www.ipcc-data.org/obs/get_30yr_means.html
	Monthly diurnal temperature range	From CRU 2.0 data set, input for the S-W model, Available at: http://www.ipcc-data.org/obs/get_30yr_means.html
	Monthly vapour pressure	From CRU 2.0 data set, input for the S-W model, Available at: http://www.ipcc-data.org/obs/get_30yr_means.html
	Monthly wind speed	From CRU 2.0 data set, input for the S-W model, Available at: http://www.ipcc-data.org/obs/get_30yr_means.html
	Monthly daylight duration	Calculated based on cloud cover, using the relationship of Doorenbos and Pruitt [14], input for the S-W model
	Monthly extraterrestrial radiation	Calculated based on the location latitude and the date in the year, using the method specified in Zhou et al. [15], input for the S-W model

Vegetation data	Monthly NDVI	GIMMS NDVI (Pinzonet al. [16]), input for the S-W model

Hydrological data	Daily observed streamflow	From Bureau of Water Resources of Quanzhou City

**Table 2:** Model parameter description.

Parameter	Description	Unit	Range	Value
*T*_0_	Saturated transmissivity	m^2^/h	0.1–200	Sand: 15 Silt: 10 Clay: 8
*m*	Decay factor of transmissivity	—	0.001–0.3	0.02
*S*_r max_	Maximum root zone storage	m	0.0001–0.8	Forest: 0.02 Grass: 0.01 Cropland: 0.01
*α*	Soil drying function parameter	—	−3–8	4
*n*	Average Manning's coefficient	—	0.001–0.4	0.06
Δ*t*	Temporal discretization of flow in a channel segment	—	1–8	2
Δ*l*	Spatial discretization of a channel segment	—	1–8	6

**Table 3:** Model performance at three streamflow stations.

Station name	Anxi		Honglai		Shilong	
Criteria	NSE	VR	NSE	VR	NSE	VR
Calibration period (2005-2006)	84.0%	96.3%	63.3%	111.0%	78.6%	110.2%
Validation period (2007–2009)	78.7%	109.3%	65.1%	97.5%	75.6%	107.2%
